# Establishment and validation of systematic prognostic nomograms in patients over 60 years of age with osteosarcoma: A multicenter external verification study

**DOI:** 10.1002/cam4.5736

**Published:** 2023-03-29

**Authors:** Zhuce Shao, JiaChen Li, Ze Liu, Shuxiong Bi

**Affiliations:** ^1^ Third Hospital of Shanxi Medical University Shanxi Bethune Hospital, Shanxi Academy of Medical Sciences, Tongji Shanxi Hospital Taiyuan China; ^2^ Department of Orthopaedics The Second Hospital of Shanxi Medical University Taiyuan China; ^3^ Shanxi Province Cancer Hospital Taiyuan China

**Keywords:** 60 years, cancer, cancer‐specific survival, nomogram, osteosarcoma, overall survival, SEER

## Abstract

**Background:**

The aim of this study was to develop and validate systematic nomograms to predict cancer specific survival (CSS) and overall survival (OS) in osteosarcoma patients aged over 60 years.

**Methods:**

We used data from the Surveillance, Epidemiology, and End Results (SEER) database and identified 982 patients with osteosarcoma over 60 years of age diagnosed between 2004 and 2015. Overall, 306 patients met the requirements for the training group. Next, we enrolled 56 patients who met the study requirements from multiple medical centers as the external validation group to validate and analyze our model. We collected all available variables and finally selected eight that were statistically associated with CSS and OS through Cox regression analysis. Integrating the identified variables, we constructed 3‐ and 5‐year OS and CSS nomograms, respectively, which were further evaluated by calculating the C‐index. A calibration curve was used to evaluate the accuracy of the model. Receiver operating characteristic (ROC) curves measured the predictive capacity of the nomograms. The Kaplan–Meier analysis was used for all patient‐based variables to explore the influence of various factors on patient survival. Finally, a decision curve analysis (DCA) curve was used to analyze whether our model would be suitable for application in clinical practice.

**Results:**

Cox regression analysis of clinical variables identified age, sex, marital status, tumor grade, tumor laterality, tumor size, M‐stage, and surgical treatment as prognostic factors. Nomograms showed good predictive capacity for OS and CSS. We calculated that the C‐index of the OS nomogram of the training population was 0.827 (95% CI 0.778–0.876), while that of the CSS nomogram was 0.722 (95% CI 0.665–0.779). The C‐index of the OS nomogram evaluated on the external validation population was 0.716 (95% CI 0.575–0.857), while that of the CSS nomogram was 0.642 (95% CI 0.50–0.788). Furthermore, the calibration curve of our prediction models indicated the nomograms could accurately predict patient outcome.

**Conclusions:**

The constructed nomogram is a useful tool for accurately predicting OS and CSS at 3 and 5 years for patients over 60 years of age with osteosarcoma and can assist clinicians in making appropriate decisions in practice.

## Graphical abstract

In this study, we used osteosarcoma patients over 60 years of age from the SEER database as the modeling population, identified statistically significant variables by means of multiple regression analyses, and used these variables to build nomograms that can accurately predict the risk of OS & CSS in osteosarcoma patients over 60 years of age. In addition, this study collected data from multiple medical centers as an external validation population to assess the accuracy and precision of the prediction models we have constructed.

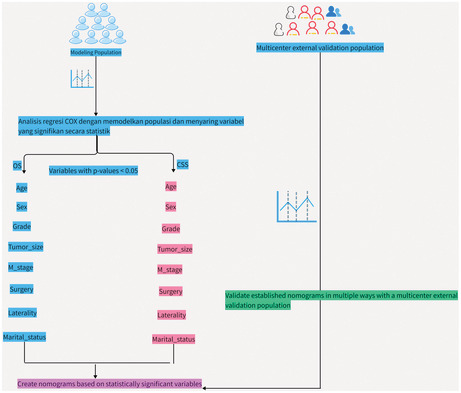

## 1 INTRODUCTION

Osteosarcoma is a malignant tumor with a poor prognosis. Its histological composition involves the mesenchymal tissue or undifferentiated connective tissue, and it is the second most common tumor after plasma cell myeloma among primary bone cancers occurring predominantly in younger and older individuals. The prognostic significance of age in patients with osteosarcoma remains to be clarified and it is generally accepted that osteosarcoma occurs with a bimodal age dispersion, with the primary incidence occurring between 10 and 20 years of age and the second highest incidence occurring after age 60 years.^1^, ^2^ Therefore, osteosarcoma patients are of great significance at age 60 years, which is not only represents the age group with the highest incidence of morbidity but also the age group with higher mortality. Given the aging population, we must consider the prognosis of osteosarcoma patients in this age group. Osteosarcoma is usually localized in the lengthy bones of the extremities close to the epiphyseal increase plate, and it especially in the distal femur, proximal tibia, and possibly in the epiphyseal plate of the proximal humerus epiphysis,^3^, ^4^ and its prognosis is very poor. Recently, the development and utilization of highly efficient chemotherapeutic agents and extended local tumor control, the incorporation of these novel therapy modalities into treatment regimens has prolonged the overall survival (OS) of patients with osteosarcoma from <20% before the 1970s to approximately 70% currently.^5^

In addition, treatment and pre/post analysis of osteosarcoma patients in recent years has revealed that the best strategy for the treatment of osteosarcoma is to introduce adjuvant chemotherapy, and then perform surgical resection of malignant areas. Adjuvant chemotherapy improves the survival rate to more than 70%,^6^ while amputation provides the local control with a survival rate of only 20%–30%.^7^ In contrast, more than half of cases treated with surgical excision of osteosarcoma alone without chemotherapy die within 12 months of diagnosis, and most pulmonary metastases occur at a median time of 10 months, providing a relatively rapid endpoint for surgery.^8^, ^9^, ^10^, ^11^ In addition, pulmonary and lymphatic metastases in patients with osteosarcoma have been the focus of attention in recent years.^12^, ^13^ Low‐grade osteosarcoma can be treated by simple surgical resection.^7^ The prognosis and survival of osteosarcoma are affected by many factors, including patient age, primary location of tumor and laterality, tumor size, and T, N, and M stage, surgery, radiotherapy, and other clinically factors. The chemotherapy regimen alone cannot be used as a treatment for osteosarcoma. Limb salvage surgery (LSS), with the addition of novel adjuvant chemotherapy is the first choice for many surgeons when treating osteosarcoma.^14^ However, if LSS cannot achieve a good therapeutic effect or is not suitable for LSS, amputation is recommended. Amputation is a more dramatic and direct method, which can immediately eliminate all parts of the tumor.^15^ The most recent techniques for the treatment of osteogenic sarcoma range from 70% to 80%, with encouraging 5‐year OS survival rates.^6^ However, previous research has identified several unbiased prognostic factors associated with survival, although no single feature can accurately predict survival of osteosarcoma patients. Consequently, there is an urgent need to establish an personalized multivariate model to accurately predict the survival and prognosis of osteosarcoma patients over 60 years of age. The Surveillance, Epidemiology, and End Results (SEER) program is a massive population‐based database for research on cancer‐related epidemiology and health‐related therapy. It gathers statistics from 18 geographic populations based in cancer registries that cover almost 30% of the US population. The nomogram is an easy to use and accurate prediction tool and has been widely applied in medical studies, mainly to evaluate cancer survival and prognosis. Nomograms contain many associated factors. It can be used to calculate the survival prognosis of individual patients by rating of the associated elements in the nomogram. Thus, the nomogram has become a reliable tool for clinical decision making and for predicting the medical outcomes of many cancers. Even though few studies have analyzed trends in the incidence and prognosis of elderly patients with osteosarcoma, no study has ever examined the factors influencing survival in patients with osteosarcoma over 60 years of age, and no study has produced a nomogram of survival in patients with osteosarcoma over 60 years of age. We have not only analyzed the factors influencing overall survival and cancer‐specific survival in patients with osteosarcoma over 60 years of age. We also created a nomogram to more visually demonstrate the risks affecting survival in patients with osteosarcoma over the age of 60.

The aim of our study was to incorporate many risk factors in the construction of two nomogram prediction models able to predict 3‐year and 5‐year survival rates of older aged patients with osteosarcoma, particularly those over 60 years of age. To achieve this goal, we evaluated the consequences of osteosarcoma patients extracted from the SEER database aged over 60 years of age from 2004 to 2015 in the form of nomograms.

## 2 MATERIALS AND METHODS

### 2.1 Study design and patient selection

Based on the SEER database, we extracted data of patients 60 years or older diagnosed with osteosarcoma from 2004 to 2015 using the SEER*STAT software (version 8.3.9.2; National Cancer Institute).

The following inclusion criteria were implemented: patients with recognized osteosarcoma registered with ICD‐O‐3/WHO morphology codes (9180–9187, 9192, 9193, 9194, 9200, 9220), (1) the first diagnosis made between 2004 and 2015, (2) adequate use of survival information, and (3) complete follow‐up records.

Cases meeting the following criteria were excluded: (1) unknown marital status, (2) unknown metastatic status, (3) unknown grade, tumor size, and T stage.

**FIGURE 1:**
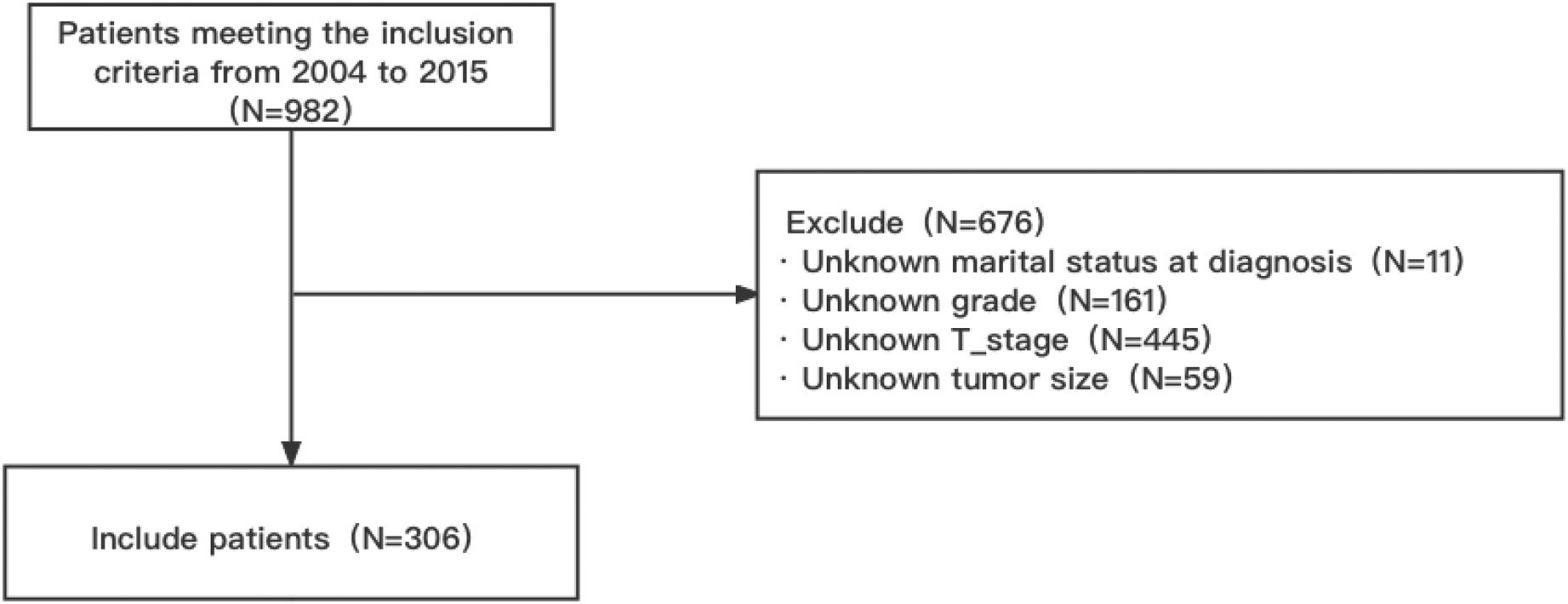
Flow chart of the study process.

A total of 306 osteosarcoma patients were included in this study. The specific flowchart including and excluding all patients is shown in Figure 1.

In addition, information on the multicenter, externally validated patient cohort was obtained from the Shanxi Cancer Hospital, the Second Hospital of Shanxi Medical University, and the Third Hospital of Shanxi Medical University.

The histological type was determined according to the International Classification of Diseases in Oncology codes. The tumor stage was established according to the seventh TNM classification of the American Joint Committee on Cancer.

This study involving human participants was reviewed and approved by the Medical Ethics Committees of the Second Hospital of Shanxi Medical University, the Third Hospital of Shanxi Medical University, and the Shanxi Cancer Hospital, respectively. In accordance with national legislation and institutional requirements, written informed consent from the legal guardians or next of kin of the participants was not required for participation in this study.

### 2.2 Data collection

The clinical and pathological information of all patients was obtained from the SEER database and included age, race, sex, marital status, tumor grade, tumor location, tumor laterality, tumor size, and stage N or M tumor, and whether or not the patient received surgical treatment, chemotherapy, or radiation therapy. Patients had two endpoint events: cancer‐specific survival (CSS) and OS.

### 2.3 Nomograms construction and verification

We designated patients diagnosed between 2004 and 2015 as the model population and patients diagnosed with osteosarcoma collected by our multiple medical centers as the external validation population. Nomograms were constructed to predict OS and CSS at 3 and 5 years incorporating many variables. The discriminative power of the nomograms was measured by receiver operating characteristic (ROC) curves and area under curve (AUC) values of survival for 3‐ and 5‐year‐OS and CSS. After establishing nomograms, we also constructed calibration plots and calculated the C‐index scores, to evaluate the predictive power of the nomograms.

### 2.4 Statistical analysis

A total of 982 patients were initially screened at the beginning of the study. After applying the inclusion and exclusion criteria, 306 patients met the study requirements.

Information on externally validated patient data for our multicenter study was initially collected from three medical centers, the Shanxi Cancer Hospital, the Second Hospital of Shanxi Medical University and the Third Hospital of Shanxi Medical University, on a total of 77 patients aged 60 years or older with osteosarcoma. After careful screening, we removed 11 patients for whom grade was not available, seven patients for whom tumor size was unknown, and three patients for whom life and death were unknown, leaving a total of 56 patients who met the requirements. We constructed nomograms for the prognosis of OS and CSS in osteosarcoma patients over 60 years based on clinical or pathological factors. Unlike previous studies that only used a few variables, we collected many variables, and finally screened out eight variables that were more meaningful than other variables through Cox regression analysis in Table 2 and Table 3. After constructing nomograms, we evaluated the predictive ability of the nomograms using the ROC curve, the AUC area, and the C‐index. An AUC > 0.9 indicated that the model has high prediction accuracy, while an AUC between 0.7 and 0.9 indicated the model had good accuracy. An AUC between 0.5 and 0.7 indicated that the model has medium and low accuracy, while an AUC < 0.5, indicated that the accuracy of the established model was very low, and the result was poor.

The C‐index was used to evaluate the accuracy of the model. We also used a calibration curve to evaluate the nomogram prediction models constructed and to evaluate the accuracy of the model. The diagonal of the calibration curve was used as the reference. This diagonal represented the optimal situation of the model, which is generally impossible to achieve. For comparison, the closer the line to 45°, the better the result; thus, the better the accuracy of the model. Moreover, using the external verification cohort, we established the prediction ability and accuracy of the model. SPSS (version 25.0) and R software (version 4.0.5, https://www.r‐project.org/) were used for statistical analysis. R software was used to develop predictive models using the “rms” package and the “surv” package to build nomograms.

## 3 RESULTS

### 3.1 Patient baseline characteristics

A total of 982 patients aged 60 years or older who were diagnosed with osteosarcoma between 2004 and 2015 were included in this study; all patients were drawn from the SEER database. Of these, 445 patients had unreported or unknown T‐stage, 220 had unknown grade or tumor size, and after excluding 11 patients with unknown marital status, 306 complete data were obtained, as shown in the exclusion flow chart in Figure 1. We also found that the clinical characteristics of the modeled population and externally validated patients were summarized, as shown in Table 1. Of the modeled patients, 156 (51%) were male and 150 (49%) were female, compared to 60.7% male and 22 (39.3%) female in the validation population. In addition, in terms of tumor grade, poorly differentiated (grades III and IV) were the most common in the modeling population at 87.3%, compared to 92.8% in the validation population. T stage was predominantly T1 and T2 in both groups, and in addition the majority of patients underwent surgery, indicating that surgery remains readily accepted. The specific clinical information and pathological characteristics of all patients are shown in Table 1.

**TABLE 1:** Baseline characteristics of the included patients.

Characteristic	Training cohort (*n* = 306)/*n* (%)	Validation cohort (*n* = 56)/*n* (%)	*p* value
Age (%)			0.342
60–69	144 (47.1)	37 (66.1)	
70–79	109 (35.6)	12 (21.4)	
>80	53 (17.3)	7 (12.5)	
Sex (%)			0.187
Male	156 (51.0)	34 (60.7)	
Female	150 (49.0)	22 (39.3)	
Grade (%)			0.112
Grade I	17 (5.6)	2 (3.6)	
Grade II	22 (7.2)	2 (3.6)	
Grade III	84 (27.5)	15 (26.7)	
Grade IV	183 (59.8)	37 (66.1)	
Laterality (%)			0.136
Left	112 (36.6)	14 (25)	
Right	120 (39.2)	19 (33.9)	
Bilateral	2 (0.7)	6 (10.7)	
Not a paired site	72 (23.5)	17 (30.4)	
Histologic_type (%)			0.666
Osteosarcoma, NOS	230 (75.2)	43 (76.8)	
Chondroblastic osteosarcoma	35 (11.4)	6 (10.7)	
Others	41 (13.4)	7 (12.5)	
T_stage (%)			0.368
T1	135 (44.1)	27 (48.2)	
T2	151 (49.3)	25 (44.6)	
T3	9 (2.9)	4 (7.2)	
TX	11 (3.6)	0 (0)	
N_stage (%)			0.214
N0	274 (89.5)	48 (85.8)	
N1	14 (4.6)	4 (7.1)	
NX	18 (5.9)	4 (7.1)	
M_stage (%)			0.563
M0	237 (77.5)	40 (71.4)	
M1	62 (20.3)	16 (28.6)	
MX	7 (2.3)	0 (0)	
Surgery (%)	235 (76.8)		0.570
Yes	235 (76.8)	42 (75.0)	
No	71 (23.2)	14 (25.0)	
Radiation (%)			0.397
Yes	84 (27.5)	17 (30.4)	
No	222 (72.5)	39 (69.6)	
Chemotherapy (%)	138 (45.1)		0.483
Yes	138 (45.1)	24 (42.9)	
No	168 (54.9)	32 (57.1)	
Tumor size (%)			0.422
<92	177 (57.8)	28 (50.0)	
92–147	84 (27.5)	17 (30.4)	
>147	45 (14.7)	11 (19.6)	
Marital status (%)			0.215
Married	191 (62.4)	32 (57.1)	
Single	115 (37.6)	24 (42.9)	
OS = *n* (%)	221 (72.2)	40 (71.4)	0.811
CSS = *n* (%)	170 (55.6)	29 (51.8)	0.459

### 3.2 Kaplan–Meier survival analysis

**FIGURE 2:**
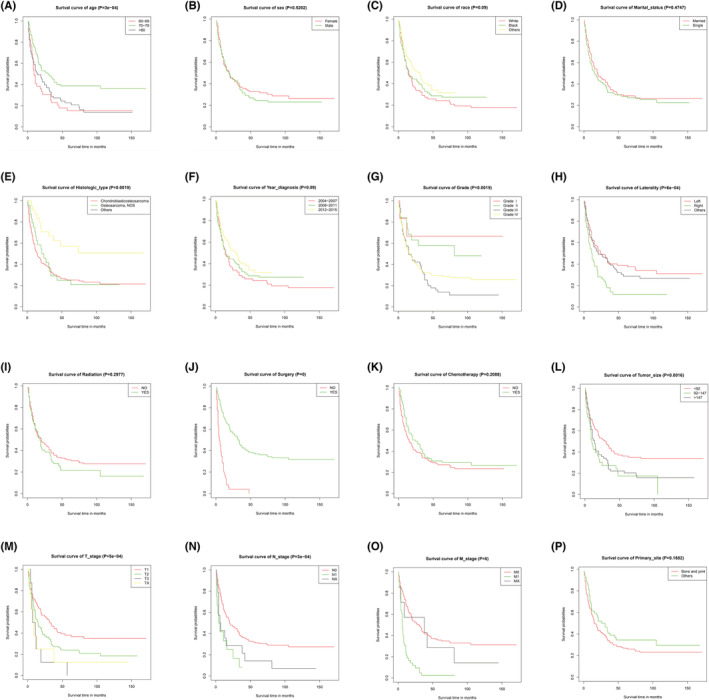
Kaplan–Meier's overall survival analysis of osteosarcoma patients over 60 years old was based on (A)–(P).

We used all clinical variables and pathological characteristics of osteosarcoma patients to calculate the outcomes using the Kaplan–Meier analysis, which was also used to analyze interesting variables not reported in the baseline data table. The specific survival curves are shown in Figure 2. The prognosis of patients aged 70–79 years was better than that of patients aged 60–69 and >80 years. The prognosis of the surgically treated patients was significantly stronger than that of those who received surgical treatment. Of these, the prognosis of grade I and II patients was better, while the prognosis of grade III and IV was inferior, which is also an important finding. Of interest, the prognosis of osteosarcoma patients who received radiotherapy or chemotherapy was similar to that of patients who do not receive therapy, and may correspond to one of the reasons for the low proportion of osteosarcoma patients willing to undergo chemotherapy or radiotherapy. Willingness to undergo therapy may be associated with the lack of obvious contribution to survival and prognosis. Kaplan–Meier findings, including the described variables, are shown in Figure 2.

### 3.3 Construction of the OS nomogram

**FIGURE 3:**
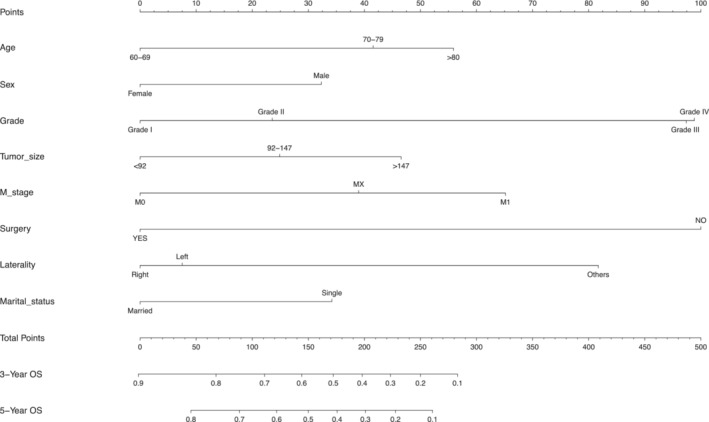
Nomogram for the prediction of 3‐ and 5‐year OS of osteosarcom of over 60 years old.

After a Cox regression analysis of the patient data, we found that the most statistically significant overall survival for osteosarcoma patients over 60 years of age was for eight variables, including age, gender, tumor grade, and size, as shown in Table 2. Therefore, we next chose these eight variables to construct a nomogram for OS. The nomograms were constructed by collecting all clinical variables and pathological characteristics of osteosarcoma patients over 60 years of age. Figure 3 shows the nomogram chart, which differs from previous studies because it is drawn using as clinical features and pathological characteristics of many variables. The objective was to achieve a personalized prediction of OS in patients over 60 years of age. Based on the nomogram, the basic, clinical and pathological characteristics of various patients on survival and prognosis are reflected by the line that indicates the total points and the corresponding single score for each factor can be obtained. Individualized scores can be determined for each patient. The individual scores corresponded to basic information, clinical, and pathological characteristics of the patient, and then the calculated total score corresponded to the percentage of line segments in the last two lines of the nomogram and indicated to the probability of prognostic survival. We found that among all the factors included, surgical treatment and tumor grade had the greatest impact on prognosis and survival rate, followed by tumor laterality and M stage. Furthermore, the existence of other factors exerted different degrees of influence on prognosis. Specifically, the predictive probability of prognosis and survival can be calculated separately, as shown in Figure 3.

**TABLE 2:** Cox regression results of OS included in patients.

Variables	Univariate analysis	*p* value	Multivariate analysis	*p* value
	HR (95%CI)		HR (95%CI)	
Age	1.47 (1.235–1.749)	1.43 E‐05	1.328 (1.089–1.619)	0.005
Sex	1.325 (1.015–1.729)	0.0382	1.425 (1.041–1.952)	0.027
Grade	1.333 (1.125–1.579)	0.001	1.376 (1.134–1.670)	0.001
T_stage	1.373 (1.163–1.622)	0.0002	0.914 (0.721–1.158)	0.456
N_stage	1.313 (1.043–1.654)	0.0205	0.924 (0.687–1.244)	0.603
M_stage	1.8 (1.441–2.249)	2.23 E‐07	1.815 (1.326–2.484)	0.0001
Tumor_size	1.401 (1.18–1.663)	0.0001	1.394 (1.110–1.750)	0.004
Surgery	0.258 (0.191–0.350)	<2 e‐16	0.300 (0.205–0.441)	8.31 E‐10
Radiation	1.115 (0.833–1.491)	0.465	0.841 (0.615–1.151)	0.28
Chemotherapy	0.947 (0.727–1.23)	0.687	0.783 (0.573–1.071)	0.126
Laterality	1.184 (1.056–1.328)	0.00377	1.313 (1.159–1.487)	1.93 E‐05
Histologic_type	0.744 (0.610–0.908)	0.00369	0.976 (0.784–1.216)	0.83
Marital_status	1.192 (0.909–1.563)	0.203	1.339 (0.990–1.809)	0.058

### 3.4 Construction of the CSS nomogram

**FIGURE 4:**
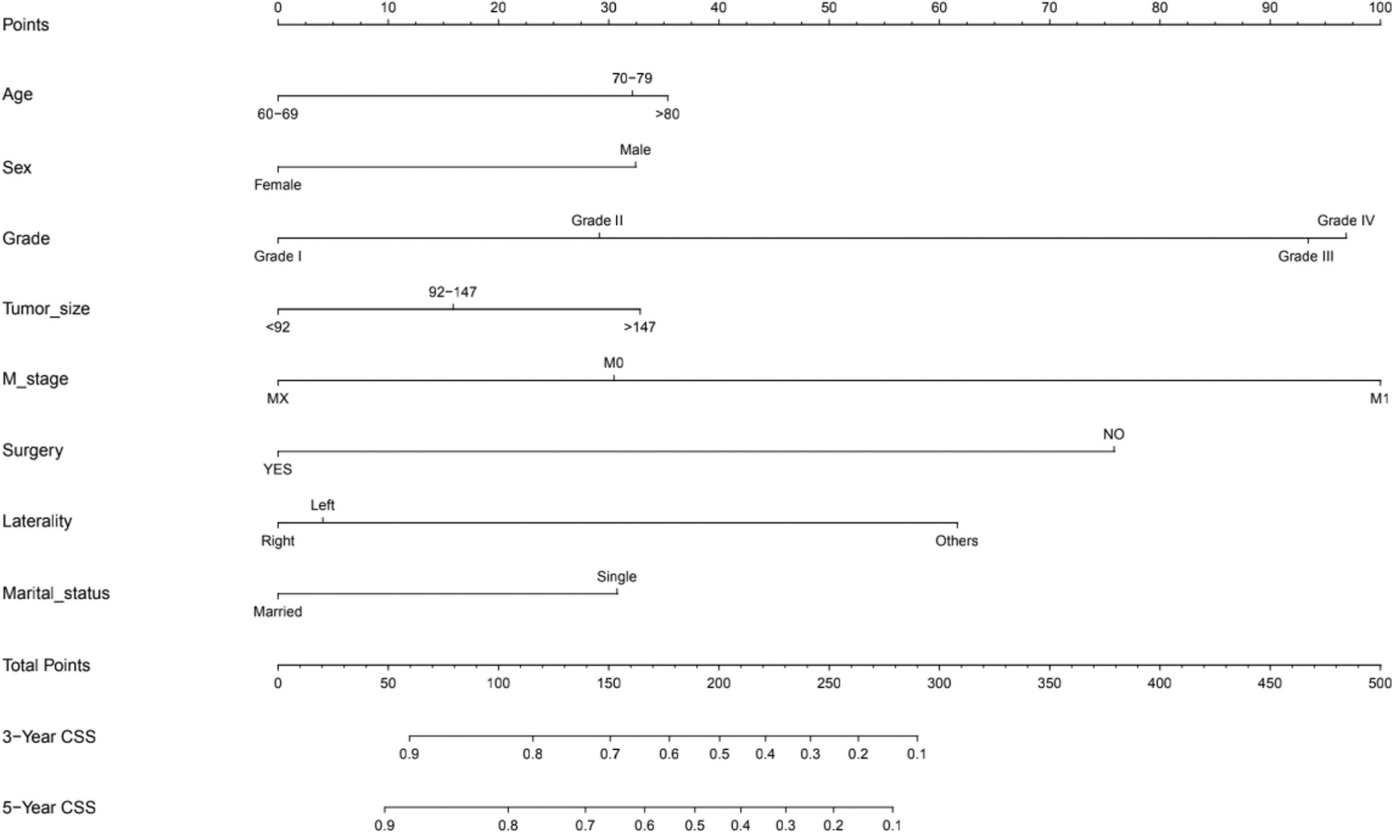
Nomogram for the prediction of 3‐ and 5‐years CSS of osteosarcom of over 60 years old.

After Cox regression analysis of the patient data, we found that the most statistically significant variables for cancer specific survival in osteosarcoma patients over 60 years of age were age, sex, grade, and size of the tumor, as shown in Table 3. Therefore, we next chose these eight variables to construct a nomogram for the CSS. A CSS nomogram was developed for osteosarcoma patients aged 60 years or older using all the variables collected (Figure 4). It was drawn by combining multiple factors including general information, clinical and pathological characteristics. In the nomogram, the combination of numbers produced a score. The influence of each factors on outcome differed; therefore, each score reflected a different outcome. Personalized scores could be determined for each individual patient. Among all factors included, M stage contributed the most to the survival results, followed by surgical treatment, and tumor laterality. Furthermore, the existence of other factors exhibited different degrees of influence on prognosis. Finally, summing the different scores of all general, clinical, and pathological factors allowed to obtain each patient's 3‐ and 5‐year prognostic probability of CSS.

**TABLE 3:** Cox regression results of CSS included in patients.

Variables	Univariate analysis	*p* value	Multivariate analysis	*p* value
	OR (95%CI)		OR (95%CI)	
Age	1.418 (1.162–1.73)	0.0006	1.344 (1.071–1.685)	0.011
Sex	1.423 (1.049–1.93)	0.0234	1.514 (1.055–2.172)	0.024
Grade	1.399 (1.144–1.71)	0.00107	1.457 (1.157–1.834)	0.001
T_stage	1.307 (1.076–1.587)	0.00687	0.839 (0.635–1.109)	0.217
N_stage	1.393 (1.082–1.793)	0.0101	1.019 (0.734–1.415)	0.91
M_stage	1.854 (1.445–2.38)	1.25 E‐06	1.854 (1.306–2.630)	0.0005
Tumor_size	1.414 (1.163–1.719)	0.000503	1.487 (1.145–1.931)	0.003
Surgery	0.253 (0.180–0.355)	2.69 E‐15	0.312 (0.202–0.481)	1.33 E‐07
Radiation	1.099 (0.788–1.531)	0.579	0.849 (0.593–1.216)	0.372
Chemotherapy	1.063 (0.787–1.437)	0.689	0.854 (0.598–1.219)	0.383
Laterality	1.146 (1.004–1.307)	0.0433	1.300 (1.125–1.502)	0.0004
Histologic_type	0.755 (0.602–0.946)	0.0148	1.002 (0.782–1.284)	0.985
Marital_status	1.194 (0.877–1.627)	0.26	1.374 (0.976–1.935)	0.069

### 3.5 Verification of the OS and CSS nomograms

**FIGURE 5:**
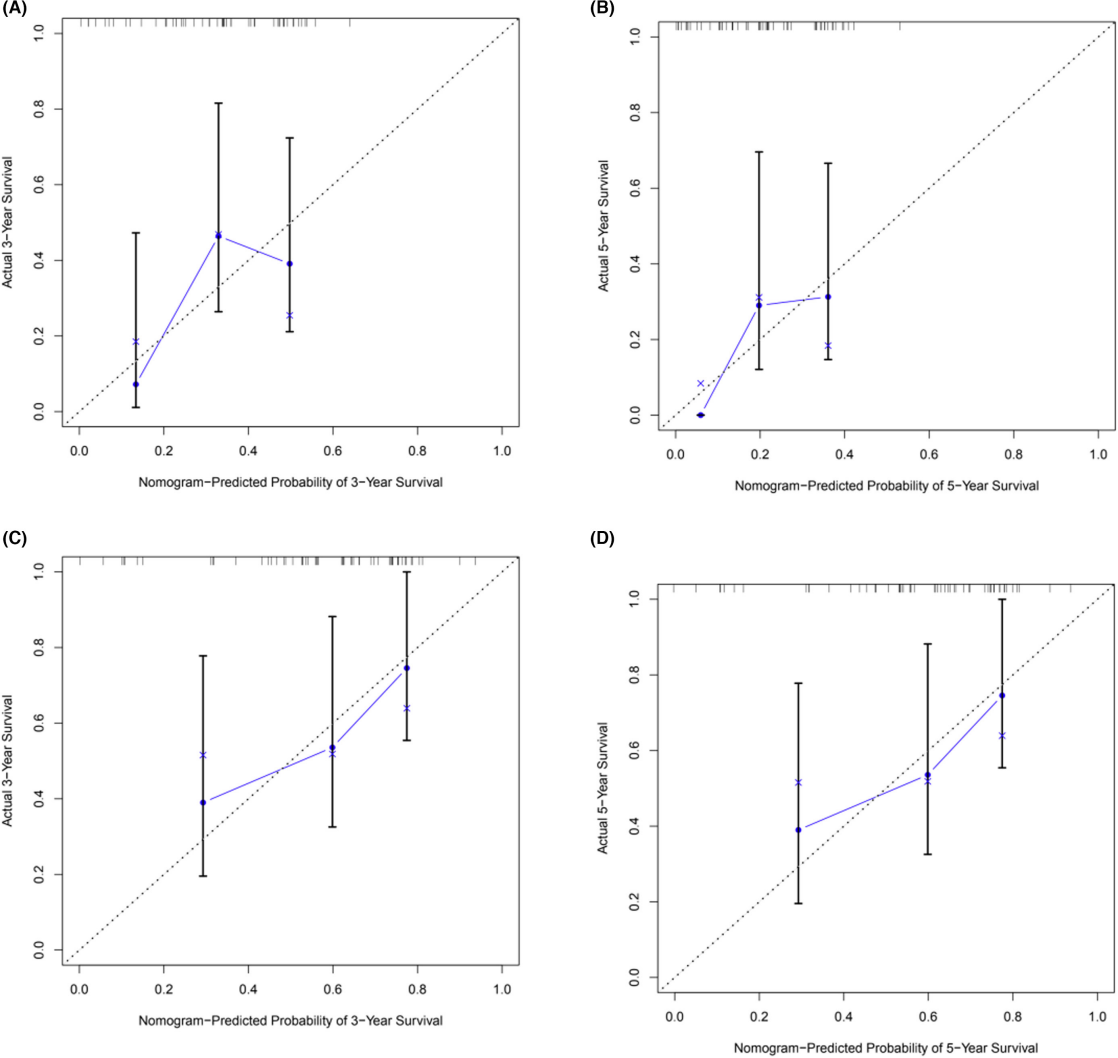
Calibration chart of nomograms have been constructed. (A) Calibration chart of external verified 3‐year OS. (B) Calibration chart of externally verified 5‐year OS. (C) Calibration chart of externally verified CSS for 3 years. (D) Calibration chart of externally verified CSS for 5 years.

**FIGURE 6:**
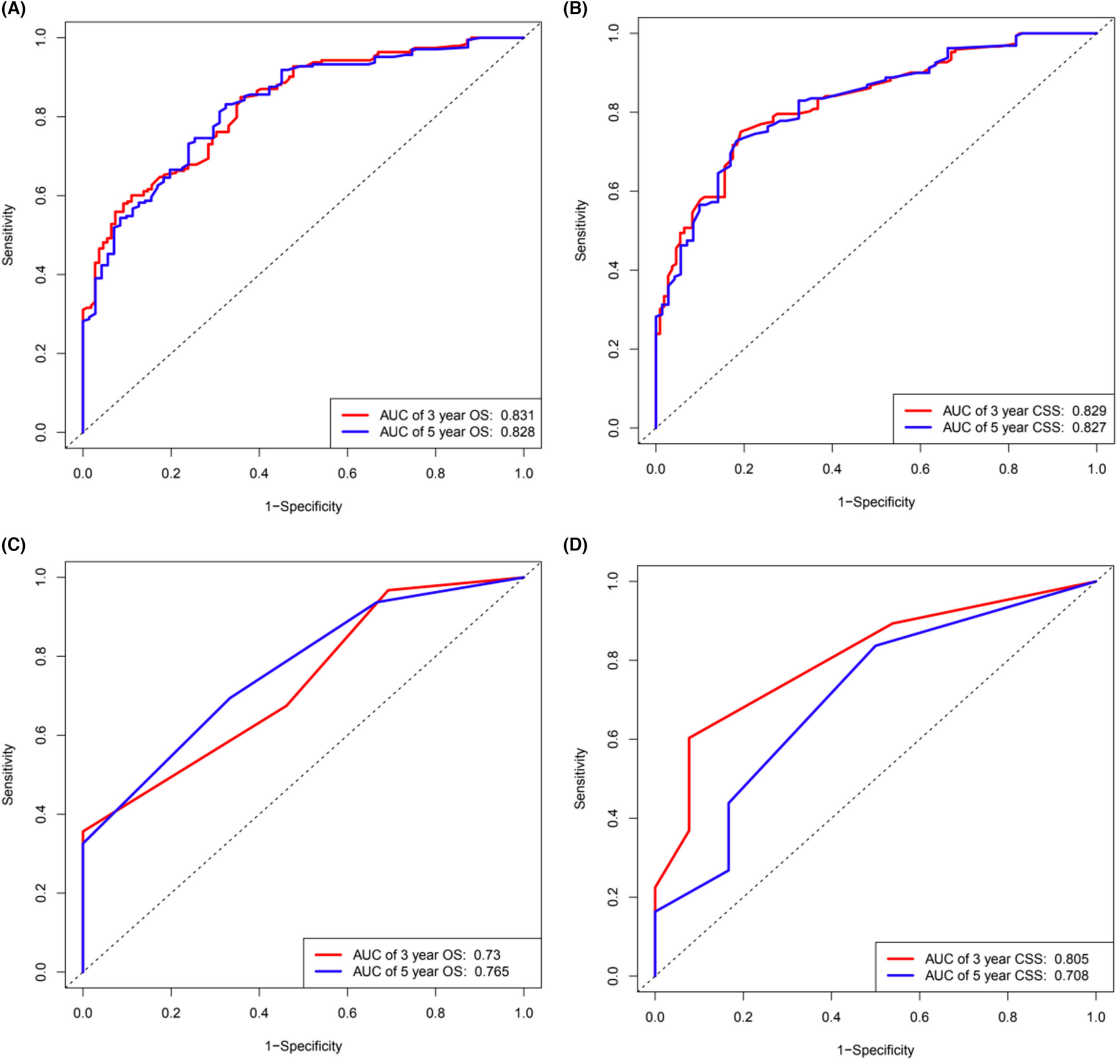
Receiver operating characteristic (ROC) of the already constructed nomograms. (A) the 3‐ and 5‐years OS of the training population. (B) the 3‐ and 5‐years CSS of the training population. (C) the 3‐ and 5‐years OS of the validation population. (D) the 3‐ and 5‐years CSS of the validation population.

ROC curves (Figure 6) were used to evaluate the predictive performance of the 3‐ and 5‐year OS for the training groups (AUC = 0.831 and AUC = 0.828, respectively) (Figure 6A) and the for external verification group (AUC = 0.730 and AUC = 0.765, respectively) (Figure 6C), the 3‐ and 5‐year CSS for the training group (AUC = 0.829 and AUC = 0.827) (Figure 6B) and for the external verification group of (AUC = 0.805 and AUC = 0.708) (Figure 6B). Overall, these findings indicate that the models were effective predictive nomograms. Figure 5 shows the calibration diagram. The calibration curves of the training group and the validation group (Figure 5) were used to verify the accuracy of the nomogram prediction results relative to the true incidence rate. When the calibration curve is diagonal, which is ideal case, the prediction probability of the calibration curve is equal to the actual probability. In general, this is not a straight diagonal line. However, the closer to the diagonal line, the better the predictive effect. Finally, we found that the calibration curve obtained from the study showed a good fit with its 45° diagonal line.

**FIGURE 7:**
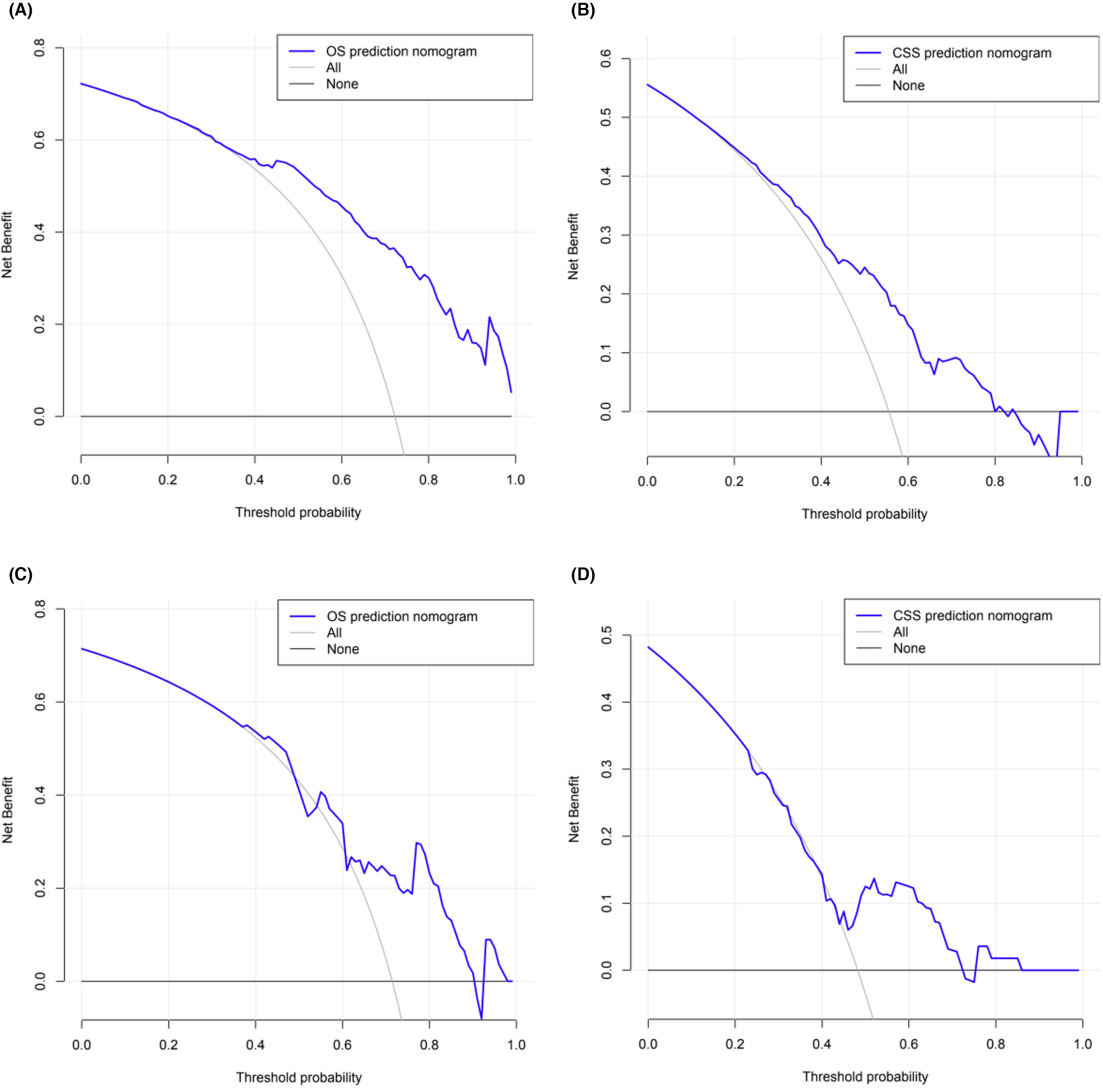
Decision curve analysis (DCA) of predictive nomograms. (A) DCA of OS modeling crowd. (B) Modeling the DCA of CSS of the population. (C) DCA of OS of external authentication crowd. (D) DCA of CSS of external validation population.

We also determined that the OS C‐index of the training population was 0.827 (95%CI 0.778–0.876), and the CSS C‐index of the training population was 0.722 (95%CI 0.665–0.779), which indicated that the accuracy of the nomogram models was very good. Figure 7 shows decision curve analysis. We also used the DCA curve to show that the nomograms calculated from the training population are of great significance and effectiveness in clinical application.

## 4 DISCUSSION

Cancer has become a global problem that cannot be ignored—is one of the most common causes of death among older aged adults, with a high mortality rate from osteosarcoma. Over 3600 new bone cancer diagnoses and 1720 deaths from bone cancer occur every year in the United States,^16^ with osteosarcoma being the predominating type. Based on the SEER database, we constructed two new nomogram tools that are easy to use and exhibited high precision in verification cohorts, indicating that they can be used to predict survival probability in terms of 3‐ and 5‐year OS and CSS of osteosarcoma patients over 60 years of age. This could be considered an improvement over a previous study that neglected to establish a predictive model for patients over 60 years of age, especially given that global aging is an increasing concern. In both the training and the validation populations, the nomograms showed very good and convincing accuracy, which indicated that the nomograms have potential clinical practical significance. No study in osteosarcoma patients has evaluated as many variables as those evaluated in the present study.

Nomograms have long been used to predict clinical diseases, especially the survival and prognosis of various cancers, mainly because nomograms can simplify the statistical prediction model into individual scores, which can be customized for individual patients, and the final score estimates the probability of the end point (such as death or metastasis). The easy‐to‐understand graphical interface of the nomogram facilitates their use when clinicians communicate with patients about treatment options or prognostic strategies and provides clinicians with more optional decision‐making information.^17^ Nomograms satisfy the need for biological and therapeutic integrated models, as well as the need for personalized treatment that can provide unique patient prognosis prediction.^18^ Validation of the nomogram before clinical application is crucial for physicians.^19^ However, the statistics used in the construction of these models must be carefully reviewed, and the uncertainty of point estimation must be verified and calibrated using different approaches. Many previous studies have developed prediction models that estimate the survival and prognosis of osteosarcoma patients. Among these, some studies established nomograms but did not distinguish between age groups.^20^, ^21^ Additionally, Zhao et al. constructed and validated a nomogram for patients with osteosarcoma in patients under 60 years of age.^22^ However, in our study, osteosarcoma patients older than 60 years were used to establish our model, which reflects the importance of stratifying patients over the age of 60 years. Furthermore, research on predictors of prognosis and survival in patients with osteosarcoma remains controversial. Therefore, considering the above reasons, our study aim was to develop and validate an easy‐to‐understand, high‐precision nomogram tool. The age range was limited to patients 60 years or older. The most important difference of our model is that we used many variables to establish the tool. To better reflect the prediction ability of the nomogram, we evaluated the nomogram in the most comprehensive way.

For many cancers, studies have long recognized age as an important factor that affects prognosis and survival^23^, ^24^ in patients with osteosarcoma. A retrospective study by Hagleitner et al. analyzed 102 osteosarcoma patients. The prognosis of older patients was relatively poor, while the survival prognosis of younger patients was much better.^25^ Furthermore, Tsuchie et al. used age at 40‐years as the cut‐off value, to evaluate the prognosis of younger and older individuals with primary osteosarcoma and determined that older patients exhibited a poorer prognosis.^26^ Our study showed that OS and CSS in patients with osteosarcoma over 60 years of age were better in those of patients 70–79 years of age.

The usefulness of chemotherapy for osteosarcoma patients is controversial. Our study also determined that OS and CSS in patients with osteosarcoma over 60 years of age receiving chemotherapy was slightly superior than those who did not receive chemotherapy, but this difference was not significant. Reasons may be related to delayed medical advice, resistance to chemotherapy, low tolerance to unstable surgery, and other metastases at diagnosis. A total of 736 patients with osteosarcoma in adolescents ineligible for chemotherapy were included in the latest study.^27^ This is also similar to our findings, although the age groups differed.

Our study showed that marital status is also an important factor affecting the survival of osteosarcoma patients—married patients having a slightly better prognosis than single patients—which is similar to the results of a study by Qiu et al.^28^ Those researchers concluded that marital status was related to survival, implying that married patients had a higher survival rate than widowed subjects, and worse prognoses of osteosarcoma. Several recent studies have confirmed a relationship between marital status and between survival performance, marital status being a predictor of OS and CSS. In most cases, the prognosis of widowed patients used to be worse than in different groups.^29^ Another study also, confirmed that the risk of death was greater in unmarried, divorced/separated, and widowed patients in contrast to the married patients.^30^ Our study further highlights how the impact of marital status on cancer is similar to that of other studies describing the close relationship between cancer and marital status.^31^, ^32^, ^33^, ^34^ The benefits of marriage for the disease may be due to the fact that both partners support each other, give each other confidence in overcoming the disease, reduce anxiety and negative thoughts, and are more willing to cooperate with treatment. Therefore, clinicians should improve awareness and decision‐making approaches for single unmarried patients.

Surgical resection of the primary tumor site plays a key role in the management of patients with osteosarcoma. In our investigation, we demonstrated favorable survival rates in older surgically resected patients. Similar to the results of other studies, surgical treatment enhanced the prognosis of the patient and survival.^35^, ^36^ The SEER database does not collect data throughout chemotherapy and surgical treatment, so we could not explore the impact of these two variables.

The improvement in patient prognosis of patients receiving chemotherapy was not significant.^27^ Based on the SEER database, this study analyzed data limited to age, race, sex, marital status, tumor grade, location of tumor, tumor laterality, tumor size and T, N, M stage, surgical treatment, chemotherapy, and radiation therapy were predictors of survival in osteosarcoma patients 60 years and older. Although there have been many studies of bone tumors in recent years,^22^, ^37^, ^38^ none have examined the survival rates of patients over 60 years of age with osteosarcoma as we have done in this study. It should be clear that nomograms cannot fully and accurately predict the prognosis and survival of all patients. Although we used ROC curve analysis and its AUC, as well as the DCA, C‐index, and calibration chart, nomograms can only be used as a tool for clinical work. Finally, a prediction model for patients with osteosarcoma warrants further research and development. In addition, SEER data does not contain variables including body mass index, alcohol, and tobacco consumption habits, which are also have a potential influence on patient prognosis and survival time, and thus, represents a study limitation. The insufficient number of patients collected is one of the limitations of our study because osteosarcoma is inherently a rare disease, and although patients over 60 years of age have the second highest incidence of osteosarcoma, the number is even rarer compared to adolescent patients. We collected multicenter validation, more using data from Chinese patients for validation. This is one of the limitations of our manuscript. But, we believe that our use of data from Chinese patients is generalizable in that we collected all consecutive patients meeting the criteria over a period of time, and although all are persons of the yellow race, there is no significant bias, and these patients are inherently highly randomized. More importantly, persons of the yellow race we used, if validated with fair results, is more indicative of better extrapolation of the model, as models are often built with blacks and whites in mind.

## 5 CONCLUSIONS

OS and CSS at 3 and 5 years were predicted in osteosarcoma patients aged over 60 years using a nomogram including a sizeable number of patient characteristics. The nomogram was shown to be accurate and reliable, and would allow patients to receive better and more personalized care and clinicians to forecast patients' survival prognosis, make more scientific judgments, and design therapy and follow‐up procedures.

## AUTHOR CONTRIBUTIONS

**Zhuce Shao:** Conceptualization (lead); data curation (lead); formal analysis (lead); investigation (lead); methodology (lead); validation (lead); visualization (lead); writing – original draft (lead); writing – review and editing (lead). **JiaChen Li:** Data curation (equal). **Ze Liu:** Data curation (equal). **Shuxiong Bi:** Conceptualization (equal).

## FUNDING INFORMATION

This research received no grant or contribution from any funding body.

## CONFLICT OF INTEREST STATEMENT

The authors say that our research was not associated with any commercial or economic interests; thus all co‐authors declare there no financial interests to report.

## Supporting information


**Figures S1–S7**.

**Tables S1–S3**.

## Data Availability

Supplementary materials in this article include the original data and research materials. Additional information can be obtained by contacting the corresponding author.

## References

[1] Ross JA , Severson RK , Swensen AR , Pollock BH , Gurney JG , Robison LL . Seasonal variations in the diagnosis of childhood cancer in the United States. Br J Cancer. 1999;81(3):549‐553. doi:10.1038/sj.bjc.6690729 10507784 PMC2362924

[2] Mirabello L , Troisi RJ , Savage SA . Osteosarcoma incidence and survival rates from 1973 to 2004: data from the surveillance, epidemiology, and end results program. Cancer. 2009;115(7):1531‐1543. doi:10.1002/cncr.24121 19197972 PMC2813207

[3] Ottaviani G , Jaffe N . The etiology of osteosarcoma. Cancer Treat Res. 2009;152:15‐32. doi:10.1007/978-1-4419-0284-9_2 20213384

[4] Marina N , Gebhardt M , Teot L , Gorlick R . Biology and therapeutic advances for pediatric osteosarcoma. Oncologist. 2004;9(4):422‐441. doi:10.1634/theoncologist.9-4-422 15266096

[5] Lamplot JD , Denduluri S , Qin J , et al. The current and future therapies for human osteosarcoma. Curr Cancer Ther Rev. 2013;9(1):55‐77. doi:10.2174/1573394711309010006 26834515 PMC4730918

[6] Luetke A , Meyers PA , Lewis I , Juergens H . Osteosarcoma treatment ‐ where do we stand? A state of the art review. Cancer Treat Rev. 2014;40(4):523‐532. doi:10.1016/j.ctrv.2013.11.006 24345772

[7] Isakoff MS , Bielack SS , Meltzer P , Gorlick R . Osteosarcoma: current treatment and a collaborative pathway to success. J Clin Oncol. 2015;33(27):3029‐3035. doi:10.1200/jco.2014.59.4895 26304877 PMC4979196

[8] Friedman MA , Carter SK . The therapy of osteogenic sarcoma: current status and thoughts for the future. J Surg Oncol. 1972;4(5):482‐510. doi:10.1002/jso.2930040512 4566220

[9] Yamamoto Y , Kanzaki R , Kanou T , et al. Long‐term outcomes and prognostic factors of pulmonary metastasectomy for osteosarcoma and soft tissue sarcoma. Int J Clin Oncol. 2019;24(7):863‐870. doi:10.1007/s10147-019-01422-0 30806840

[10] Sweetnam R , Knowelden J , Seddon H . Bone sarcoma: treatment by irradiation, amputation, or a combination of the two. Br Med J. 1971;2(5758):363‐367. doi:10.1136/bmj.2.5758.363 4995895 PMC1795781

[11] Ramanujan V , Krishnamurthy A , Venkataramani K , Kumar C . Pulmonary metastasectomy in primary extremity osteosarcoma: choosing wisely, along with a brief review of literature. Indian J Cancer. 2020;57(2):172‐181. doi:10.4103/ijc.IJC_497_18 32445321

[12] Li W , Liu Y , Liu W , et al. Machine learning‐based prediction of lymph node metastasis among osteosarcoma patients. Front Oncol. 2022;12:797103. doi:10.3389/fonc.2022.797103 35515104 PMC9067126

[13] Li W , Dong S , Wang H , et al. Risk analysis of pulmonary metastasis of chondrosarcoma by establishing and validating a new clinical prediction model: a clinical study based on Seer database. BMC Musculoskelet Disord. 2021;22(1):529. doi:10.1186/s12891-021-04414-2 34107945 PMC8191035

[14] Harrison DJ , Geller DS , Gill JD , Lewis VO , Gorlick R . Current and future therapeutic approaches for osteosarcoma. Expert Rev Anticancer Ther. 2018;18(1):39‐50. doi:10.1080/14737140.2018.1413939 29210294

[15] Godley K , Watts AC , Robb JE . Pathological femoral fracture caused by primary bone tumour: a population‐based study. Scott Med J. 2011;56(1):5‐9. doi:10.1258/smj.2010.010006 21515523

[16] Siegel RL , Miller KD , Jemal A . Cancer statistics, 2020. CA Cancer J Clin. 2020;70(1):7‐30. doi:10.3322/caac.21590 31912902

[17] Iasonos A , Schrag D , Raj GV , Panageas KS . How to build and interpret a nomogram for cancer prognosis. J Clin Oncol. 2008;26(8):1364‐1370. doi:10.1200/jco.2007.12.9791 18323559

[18] Balachandran VP , Gonen M , Smith JJ , DeMatteo RP . Nomograms in oncology: more than meets the eye. Lancet Oncol. 2015;16(4):e173‐e180. doi:10.1016/s1470-2045(14)71116-7 25846097 PMC4465353

[19] Ohori Tatsuo G , Riu Hamada M , Gondo T , Hamada R . Nomogram as predictive model in clinical practice. Gan to Kagaku Ryoho. 2009;36(6):901‐906. 19542708

[20] Yang QK , Lai QY , Wang Y , Wang Y , Yao ZX , Zhang XJ . Establishment and validation of prognostic nomograms to predict overall survival and cancer‐specific survival for patients with osteosarcoma. Neoplasma. 2021;68(2):434‐446. doi:10.4149/neo_2020_200617N639 33118831

[21] Zheng W , Huang Y , Chen H , et al. Nomogram application to predict overall and cancer‐specific survival in osteosarcoma. Cancer Manag Res. 2018;10:5439‐5450. doi:10.2147/cmar.S177945 30519092 PMC6235004

[22] Zhao J , Jiao J , Su Y , Mu L . Development and validation of a nomogram for specific survival in osteosarcoma patients less than 60 years old: a population‐based study. J BUON. 2021;26(5):2097‐2105. 34761622

[23] Sun HH , Chen XY , Cui JQ , Zhou ZM , Guo KJ . Prognostic factors to survival of patients with chondroblastic osteosarcoma. Medicine (Baltimore). 2018;97(39):e12636. doi:10.1097/md.0000000000012636 30278586 PMC6181520

[24] Chung M , Chang HR , Bland KI , Wanebo HJ . Younger women with breast carcinoma have a poorer prognosis than older women. Cancer. 1996;77(1):97‐103. doi:10.1002/(sici)1097-0142(19960101)77:1<97::Aid-cncr16>3.0.Co;2-3 8630946

[25] Hagleitner MM , Hoogerbrugge PM , van der Graaf WT , Flucke U , Schreuder HW , te Loo DM . Age as prognostic factor in patients with osteosarcoma. Bone. 2011;49(6):1173‐1177. doi:10.1016/j.bone.2011.08.014 21893224

[26] Tsuchie H , Emori M , Nagasawa H , et al. Prognosis of primary osteosarcoma in elderly patients: a comparison between young and elderly patients. Med Princ Pract. 2019;28(5):425‐431. doi:10.1159/000500404 30991396 PMC6771043

[27] Jiang Y , Wang T , Wei Z . Construction and validation of nomograms for predicting the prognosis of juvenile osteosarcoma: a real‐world analysis in the Seer database. Technol Cancer Res Treat. 2020;19:1533033820947718. doi:10.1177/1533033820947718 33054584 PMC7570778

[28] Qiu S , Tao L , Zhu Y . Marital status and survival in osteosarcoma patients: an analysis of the surveillance, epidemiology, and end results (Seer) database. Med Sci Monit. 2019;25:8190‐8203. doi:10.12659/msm.918048 31672959 PMC6849371

[29] Wang X , Li X , Su S , Liu M . Marital status and survival in epithelial ovarian cancer patients: a seer‐based study. Oncotarget. 2017;8(51):89040‐89054. doi:10.18632/oncotarget.21648 29179497 PMC5687667

[30] Du L , Kim JJ , Chen B , Zhu S , Dai N . Marital status is associated with superior survival in patients with esophageal cancer: a surveillance, epidemiology, and end results study. Oncotarget. 2017;8(56):95965‐95972. doi:10.18632/oncotarget.21609 29221179 PMC5707073

[31] Wu C , Chen P , Qian JJ , et al. Effect of marital status on the survival of patients with hepatocellular carcinoma treated with surgical resection: an analysis of 13,408 patients in the surveillance, epidemiology, and end results (Seer) database. Oncotarget. 2016;7(48):79442‐79452. doi:10.18632/oncotarget.12722 27769053 PMC5346726

[32] Aizer AA , Chen MH , McCarthy EP , et al. Marital status and survival in patients with cancer. J Clin Oncol. 2013;31(31):3869‐3876. doi:10.1200/jco.2013.49.6489 24062405 PMC4878087

[33] Shi RL , Chen Q , Yang Z , et al. Marital status independently predicts gastric cancer survival after surgical resection–an analysis of the SEER database. Oncotarget. 2016;7(11):13228‐13235. doi:10.18632/oncotarget.7107 26840093 PMC4914354

[34] Wang XD , Qian JJ , Bai DS , Li ZN , Jiang GQ , Yao J . Marital status independently predicts pancreatic cancer survival in patients treated with surgical resection: an analysis of the seer database. Oncotarget. 2016;7(17):24880‐24887. doi:10.18632/oncotarget.8467 27036036 PMC5029750

[35] Mukherjee D , Chaichana KL , Parker SL , Gokaslan ZL , McGirt MJ . Association of Surgical Resection and Survival in patients with malignant primary osseous spinal neoplasms from the surveillance, epidemiology, and end results (SEER) database. Eur Spine J. 2013;22(6):1375‐1382. doi:10.1007/s00586-012-2621-4 23263168 PMC3676564

[36] Whelan JS , Davis LE . Osteosarcoma, chondrosarcoma, and chordoma. J Clin Oncol. 2018;36(2):188‐193. doi:10.1200/jco.2017.75.1743 29220289

[37] Li W , Liu W , Hussain Memon F , et al. An external‐validated prediction model to predict lung metastasis among osteosarcoma: a multicenter analysis based on machine learning. Comput Intell Neurosci. 2022;2022:2220527. doi:10.1155/2022/2220527 35571720 PMC9106476

[38] Liu X , He S , Yao X , Hu T . Development and validation of prognostic nomograms for elderly patients with osteosarcoma. Int J Gen Med. 2021;14:5581‐5591. doi:10.2147/ijgm.S331623 34548809 PMC8449646

