# Supplementary figures and images for: Establishment and validation of systematic prognostic nomograms in patients over 60 years of age with osteosarcoma: A multicenter external verification study
Source: Cancer Med. 2023 Mar 29;12(8):9589–603. doi: 10.1002/cam4.5736 (PMC10166929; doi:10.1002/cam4.5736)

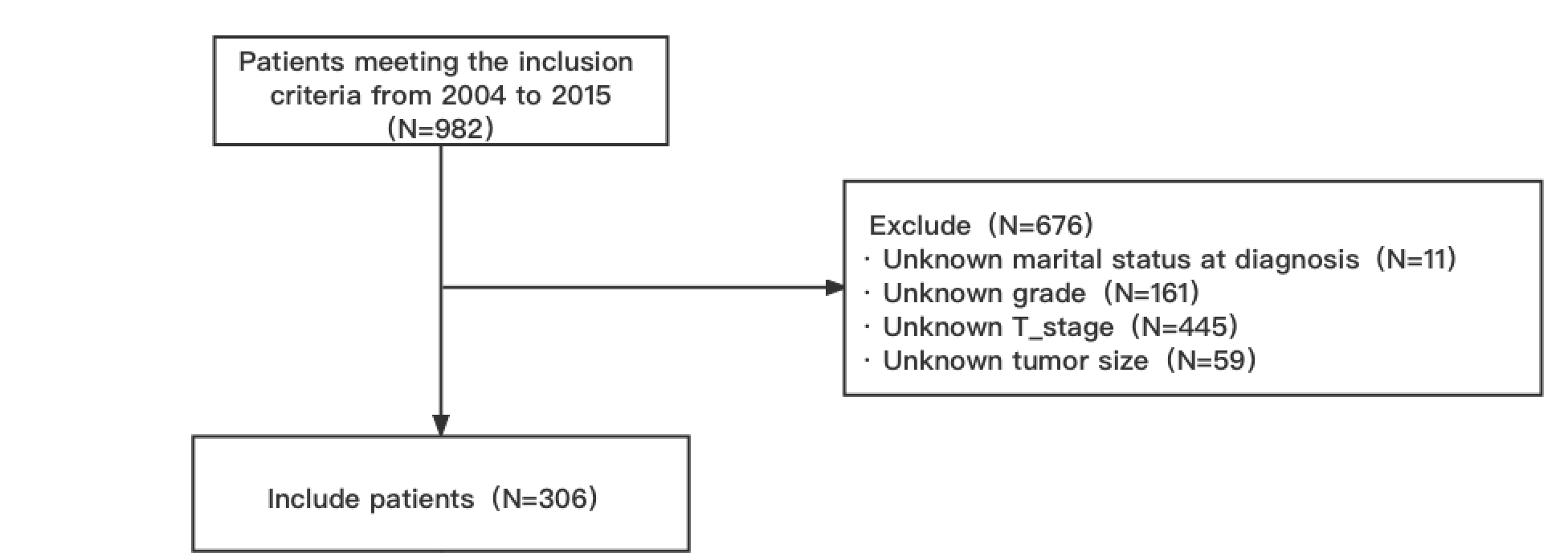


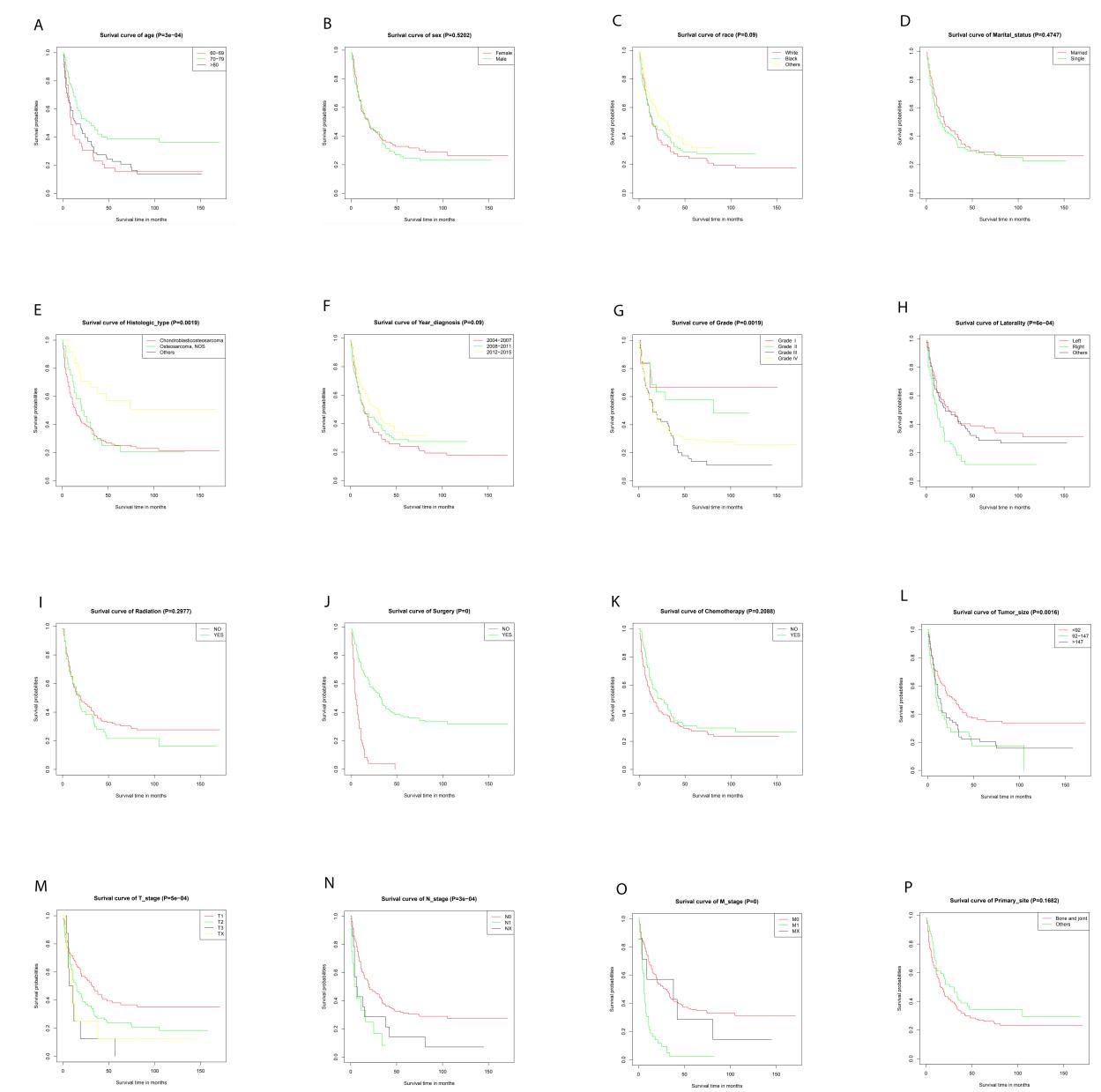


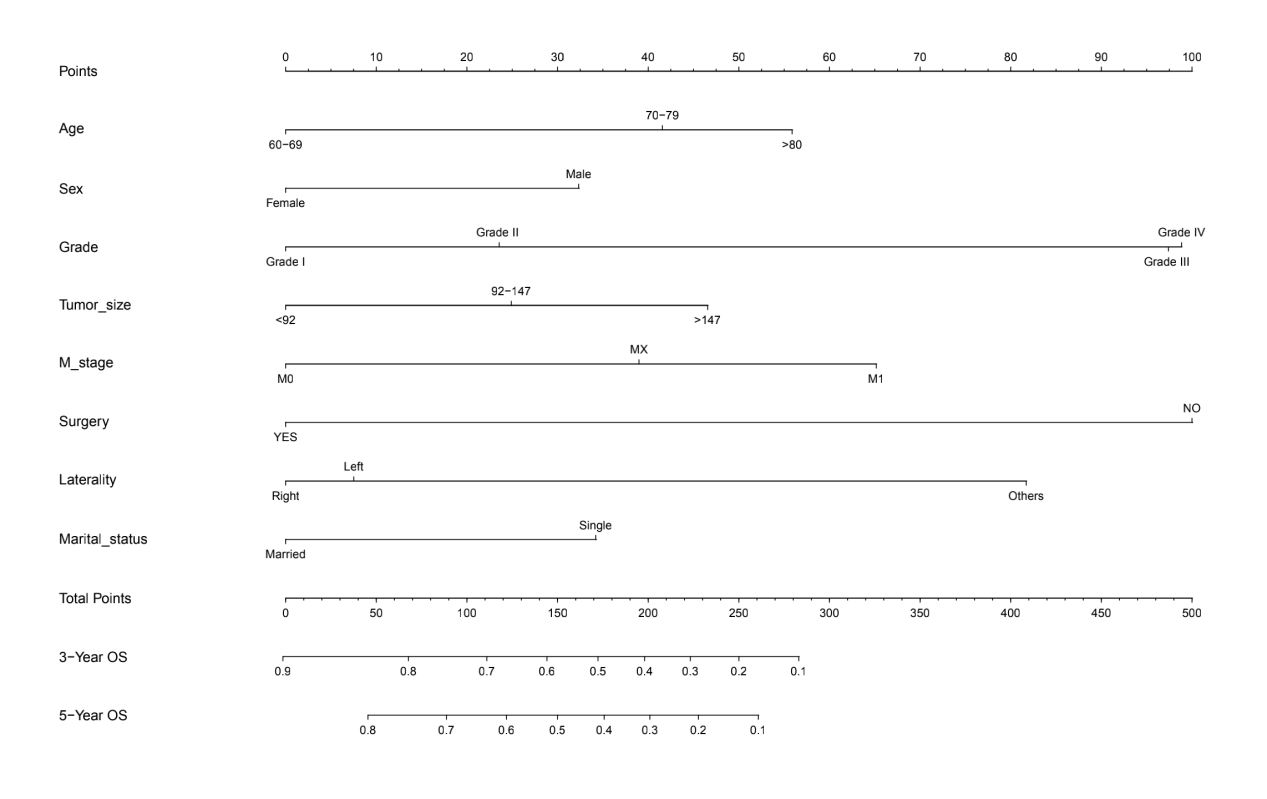


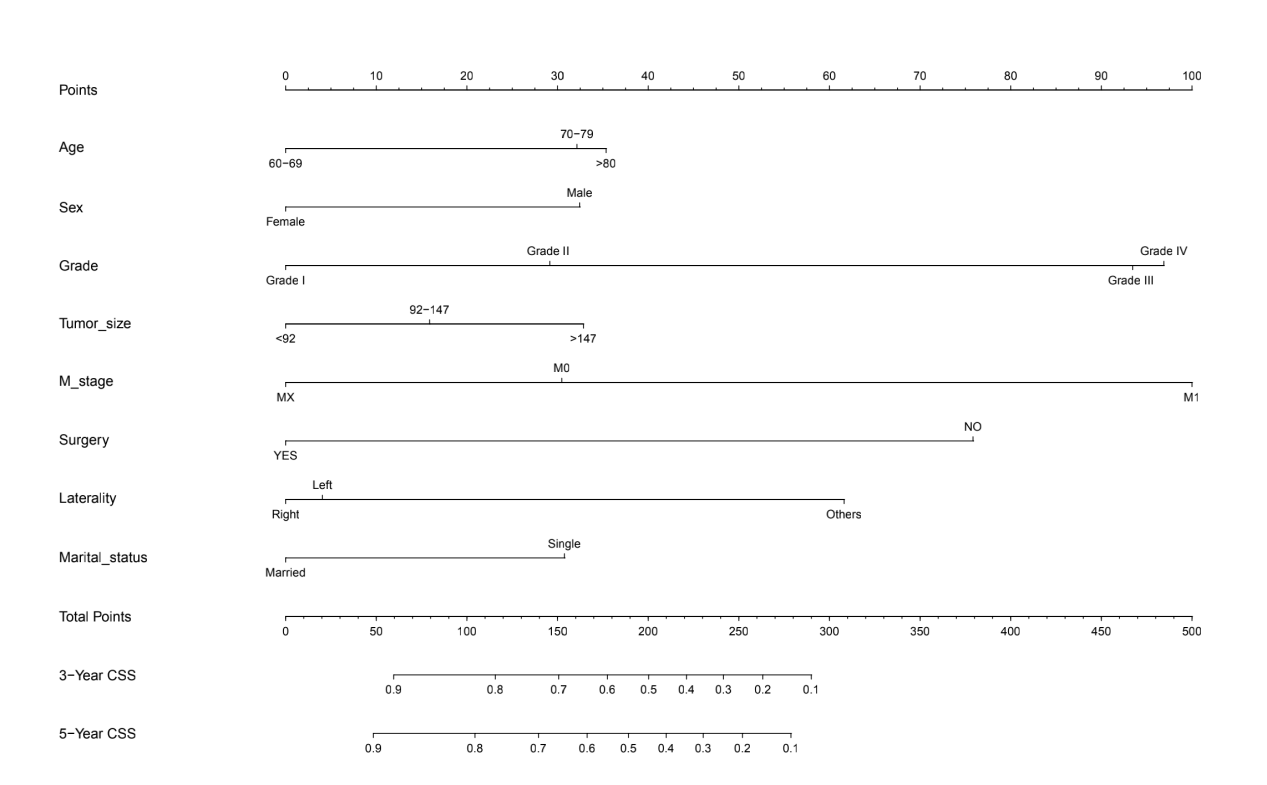


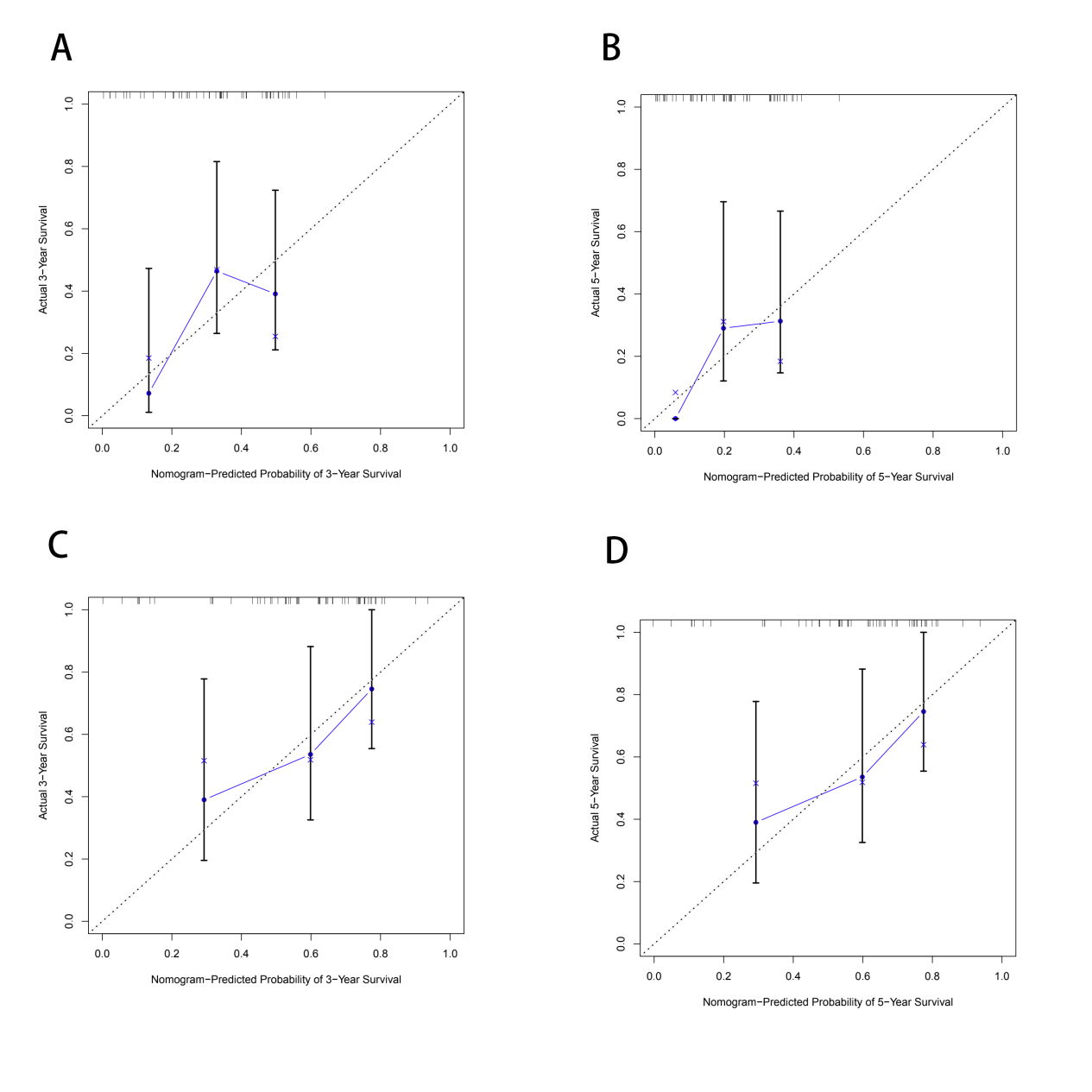


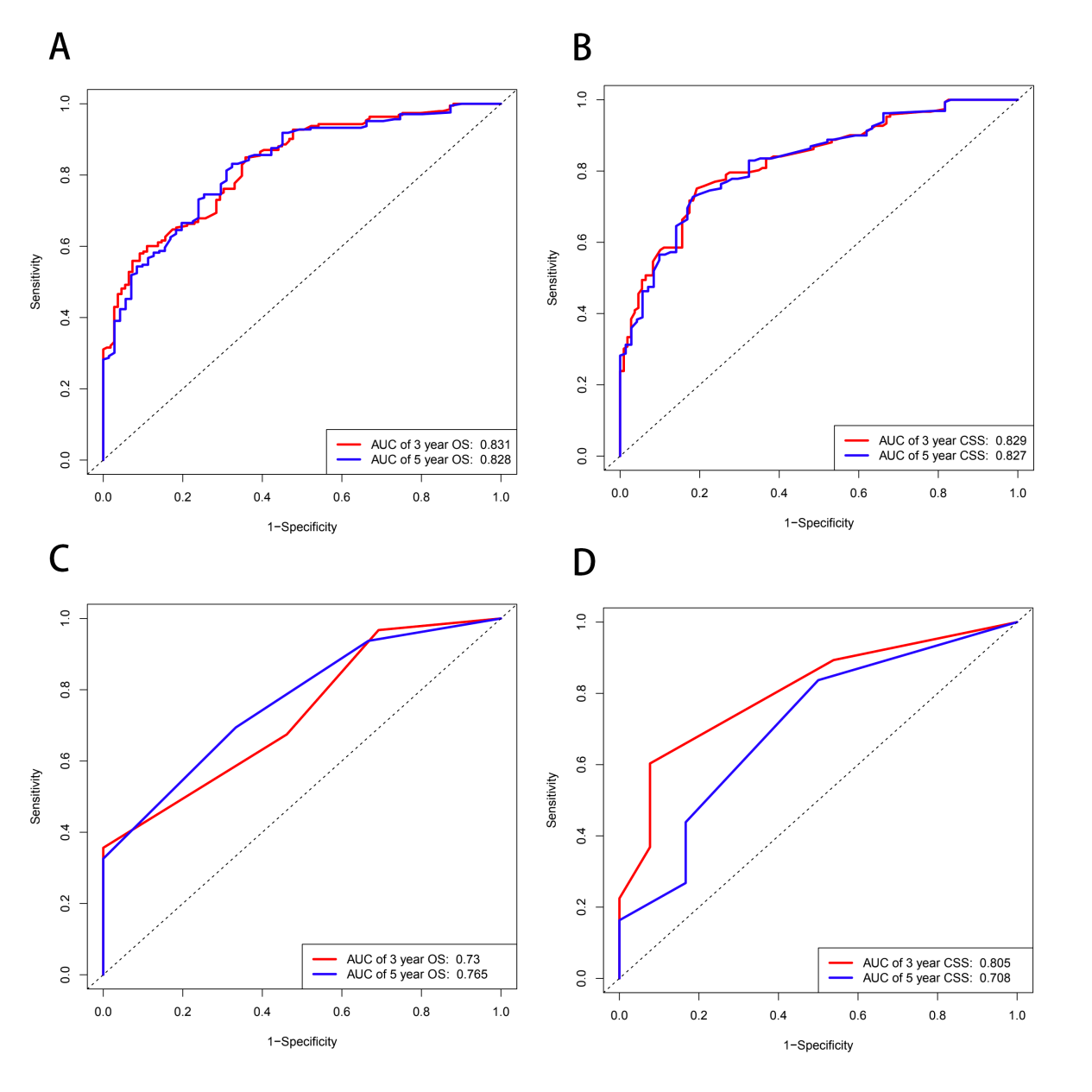


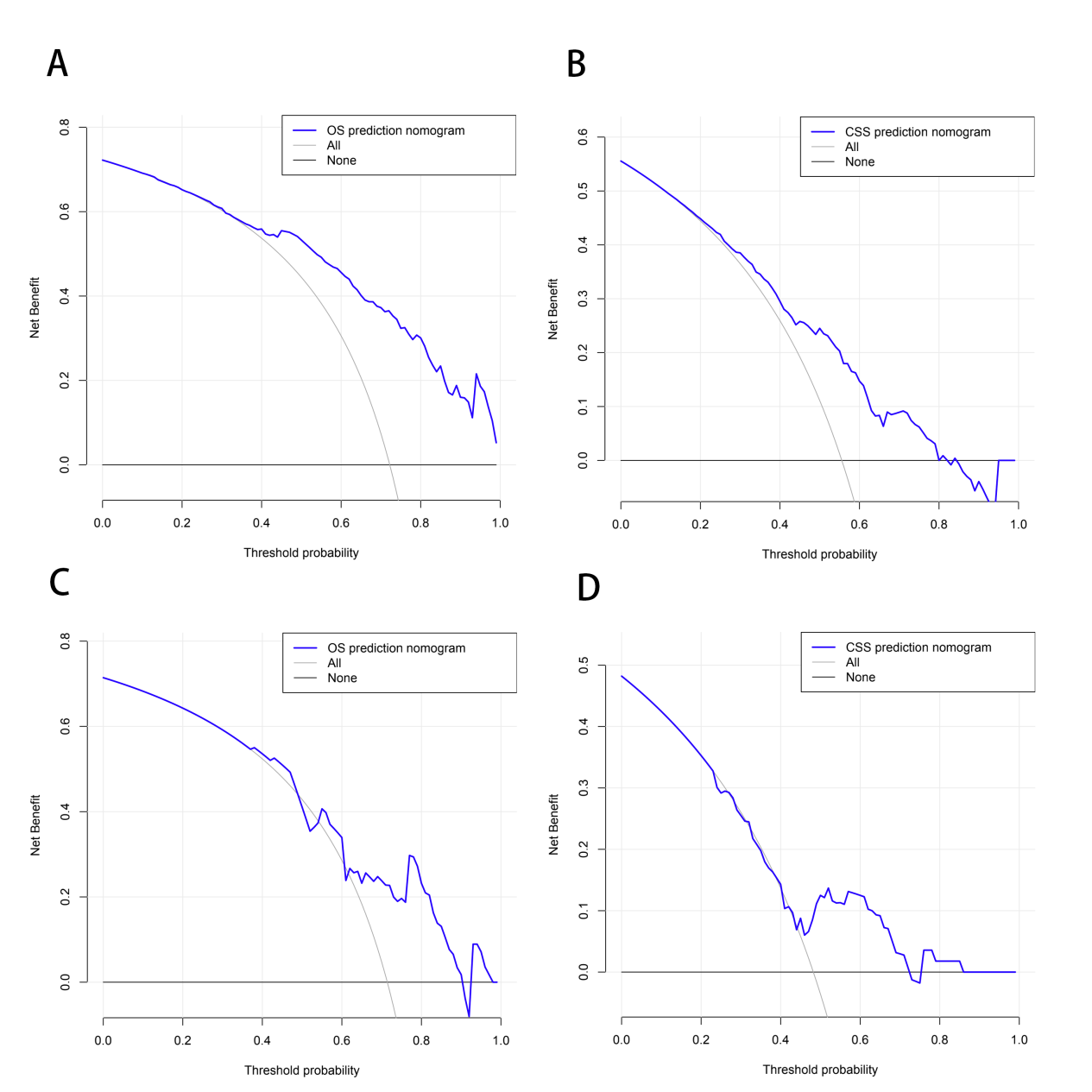

Supplement: Supplementary file 1 — Figures S1–S7. [file CAM4-12-9589-s001.docx]
