# Supplementary material for: Establishment and validation of systematic prognostic nomograms in patients over 60 years of age with osteosarcoma: A multicenter external verification study
Source: Cancer Med. 2023 Mar 29;12(8):9589–603. doi: 10.1002/cam4.5736 (PMC10166929; doi:10.1002/cam4.5736)
Supplement: Supplementary file 2 — Tables S1–S3. [file CAM4-12-9589-s002.docx]

## Tables 1 Baseline characteristics of the included patients

| **Characteristic** | **Training cohort (n=306)**  **/n (%)** | **Validation cohort (n=56)**  **/n (%)** | **P value** |
| --- | --- | --- | --- |
| Age (%) |  |  | 0.342 |
| 60-69 | 144 (47.1) | 37(66.1) |  |
| 70-79 | 109 (35.6) | 12(21.4) |  |
| >80 | 53 (17.3) | 7(12.5) |  |
| Sex(%) |  |  | 0.187 |
| Male | 156 (51.0) | 34(60.7) |  |
| Female | 150 (49.0) | 22(39.3) |  |
| Grade (%) |  |  | 0.112 |
| Grade I | 17 (5.6) | 2(3.6) |  |
| Grade II | 22 (7.2) | 2(3.6) |  |
| Grade III | 84 (27.5) | 15(26.7) |  |
| Grade IV | 183 (59.8) | 37(66.1) |  |
| Laterality (%) |  |  | 0.136 |
| Left | 112 (36.6) | 14(25) |  |
| Right | 120 (39.2) | 19(33.9) |  |
| Bilateral | 2 (0.7) | 6(10.7) |  |
| Not a paired site | 72 (23.5) | 17(30.4) |  |
| Histologic_type (%) |  |  | 0.666 |
| Osteosarcoma, NOS | 230 (75.2) | 43(76.8) |  |
| Chondroblastic osteosarcoma | 35 (11.4) | 6(10.7) |  |
| Others | 41 (13.4) | 7(12.5) |  |
| T_stage (%) |  |  | 0.368 |
| T1 | 135 (44.1) | 27(48.2) |  |
| T2 | 151 (49.3) | 25(44.6) |  |
| T3 | 9 (2.9) | 4(7.2) |  |
| TX | 11 (3.6) | 0(0) |  |
| N_stage (%) |  |  | 0.214 |
| N0 | 274 (89.5) | 48(85.8) |  |
| N1 | 14 (4.6) | 4(7.1) |  |
| NX | 18 (5.9) | 4(7.1) |  |
| M_stage (%) |  |  | 0.563 |
| M0 | 237 (77.5) | 40(71.4) |  |
| M1 | 62 (20.3) | 16(28.6) |  |
| MX | 7 (2.3) | 0(0) |  |
| Surgery(%) | 235 (76.8) |  | 0.570 |
| YES | 235 (76.8) | 42(75.0) |  |
| NO | 71(23.2) | 14(25.0) |  |
| Radiation(%) |  |  | 0.397 |
| YES | 84 (27.5) | 17(30.4) |  |
| NO | 222(72.5) | 39(69.6) |  |
| Chemotherapy(%) | 138 (45.1) |  | 0.483 |
| YES | 138 (45.1) | 24(42.9) |  |
| NO | 168 (54.9) | 32(57.1) |  |
| Tumor size (%) |  |  | 0.422 |
| <92 | 177 (57.8) | 28(50.0) |  |
| 92-147 | 84 (27.5) | 17(30.4) |  |
| >147 | 45 (14.7) | 11(19.6) |  |
| Marital status(%) |  |  | 0.215 |
| Married | 191 (62.4) | 32(57.1) |  |
| single | 115 (37.6) | 24(42.9) |  |
| OS = n (%) | 221 (72.2) | 40(71.4) | 0.811 |
| CSS = n (%) | 170 (55.6) | 29(51.8) | 0.459 |

**Tables 2 Cox regression results of OS included in patients**

| **Variables** | **Univariate analysis** | **P value** | **Multivariate analysis** | **P value** |
| --- | --- | --- | --- | --- |
|  | **HR (95%CI)** |  | **HR (95%CI)** |  |
| Age | 1.47(1.235-1.749) | 1.43E-05 | 1.328(1.089-1.619) | 0.005 |
| Sex | 1.325(1.015-1.729) | 0.0382 | 1.425(1.041-1.952) | 0.027 |
| Grade | 1.333(1.125-1.579) | 0.001 | 1.376(1.134-1.670) | 0.001 |
| T_stage | 1.373(1.163-1.622) | 0.0002 | 0.914(0.721-1.158) | 0.456 |
| N_stage | 1.313(1.043-1.654) | 0.0205 | 0.924(0.687-1.244) | 0.603 |
| M_stage | 1.8(1.441-2.249) | 2.23E-07 | 1.815(1.326-2.484) | 0.0001 |
| Tumor_size | 1.401(1.18-1.663) | 0.0001 | 1.394(1.110-1.750) | 0.004 |
| Surgery | 0.258(0.191-0.350 ) | <2e-16 | 0.300(0.205-0.441) | 8.31E-10 |
| Radiation | 1.115(0.833-1.491) | 0.465 | 0.841(0.615-1.151) | 0.28 |
| Chemotherapy | 0.947(0.727-1.23) | 0.687 | 0.783(0.573-1.071) | 0.126 |
| Laterality | 1.184(1.056-1.328) | 0.00377 | 1.313(1.159-1.487) | 1.93E-05 |
| Histologic_type | 0.744(0.610-0.908) | 0.00369 | 0.976(0.784-1.216) | 0.83 |
| Marital_status | 1.192(0.909-1.563) | 0.203 | 1.339(0.990-1.809) | 0.058 |

**Tables 3 Cox regression results of CSS included in patients**

| **Variables** | **Univariate analysis** | **P value** | **Multivariate analysis** | **P value** |
| --- | --- | --- | --- | --- |
|  | **OR (95%CI)** |  | **OR (95%CI)** |  |
| Age | 1.418(1.162-1.73) | 0.0006 | 1.344(1.071-1.685) | 0.011 |
| Sex | 1.423(1.049-1.93) | 0.0234 | 1.514(1.055-2.172) | 0.024 |
| Grade | 1.399(1.144-1.71) | 0.00107 | 1.457(1.157-1.834) | 0.001 |
| T_stage | 1.307(1.076-1.587) | 0.00687 | 0.839(0.635-1.109) | 0.217 |
| N_stage | 1.393(1.082-1.793) | 0.0101 | 1.019(0.734-1.415) | 0.91 |
| M_stage | 1.854(1.445-2.38) | 1.25E-06 | 1.854(1.306-2.630) | 0.0005 |
| Tumor_size | 1.414(1.163-1.719) | 0.000503 | 1.487(1.145-1.931) | 0.003 |
| Surgery | 0.253(0.180-0.355) | 2.69E-15 | 0.312(0.202-0.481) | 1.33E-07 |
| Radiation | 1.099(0.788-1.531) | 0.579 | 0.849(0.593-1.216) | 0.372 |
| Chemotherapy | 1.063(0.787-1.437) | 0.689 | 0.854(0.598-1.219) | 0.383 |
| Laterality | 1.146(1.004-1.307) | 0.0433 | 1.300(1.125-1.502) | 0.0004 |
| Histologic_type | 0.755(0.602-0.946) | 0.0148 | 1.002(0.782-1.284) | 0.985 |
| Marital_status | 1.194(0.877-1.627) | 0.26 | 1.374(0.976-1.935) | 0.069 |
